# Multivariate analyses of Aurignacian and Gravettian personal ornaments support cultural continuity in the Early Upper Palaeolithic

**DOI:** 10.1371/journal.pone.0323148

**Published:** 2025-06-04

**Authors:** Francesco d’Errico, Jack Baker, Daniel Pereira, Esteban Álvarez-Fernández, Martina Lázničková-Galetová, Solange Rigaud

**Affiliations:** 1 CNRS UMR 5199 De la Préhistoire à l’Actuel: Culture, Environnement, et Anthropologie (PACEA), Université Bordeaux, Talence, France; 2 SFF Centre for Early Sapiens Behaviour (SapienCE), University of Bergen, Bergen, Norway; 3 BonesLab, Dipartimento di Beni Culturali, Università di Bologna, Ravenna, Italy; 4 GIR Prehusal, Dpto. de Prehistoria, Historia Antigua y Arqueología, Facultad de Geografía e Historia, Universidad de Salamanca, Salamanca, Spain; 5 Moravian Museum, Brno, Czech Republic; 6 UMR 7194 HNHP, Equipe NOMADE, CNRS/ Muséum national d’histoire naturelle (MNHN)/ UPVD Institut de Paléontologie Humaine, Paris, France; Universita degli Studi di Ferrara, ITALY

## Abstract

Traditionally, lithic artefacts have served as the principal proxy for the definition of archaeological cultures in the Upper Paleolithic. However, the culture-historical framework in use, constructed unsystematically and shaped by regional research traditions, features a number of widely acknowledged drawbacks. Here we use personal ornaments to explore the nature of Early Upper Paleolithic cultural entities and establish to what extent they represent distinct or evolving cultural adaptations. We present an analysis of an updated georeferenced dataset composed of personal ornaments coming from two key successive Upper Paleolithic technocomplexes, the Aurignacian (42–34,000 years ago) and the Gravettian (34–24,000 years ago). Using a range of multivariate statistics, we demonstrate that, at both European and regional scales, people belonging to these technocomplexes wore similar personal ornaments, though fully-shaped personal ornaments appear more different between technocomplexes. We additionally show that the variability of the Aurignacian ornaments suggests more fragmented cultural clusters compared to the Gravettian, implying more extensive symbolic networks in the latter. Despite a long-standing consensus based on other archaeological proxies, which emphasises the dissimilarity between these cultural entities, our results demonstrate the complex nature of Upper Paleolithic cultures which are characterised by discontinuities in economic and technical systems and continuity in the culturalisation of the body.

## Introduction

One of the persistent challenges in prehistoric archaeology is determining how past social structures and identities were expressed in the material record. Traditional approaches have often relied on typological classifications of material culture, assuming that artifacts such as lithic tools can serve as indicators of distinct social or linguistic groups (e.g., [1]). However, this culture-history paradigm—which frames material culture as a proxy for bounded social entities—has faced increasing criticism for its rigidity, its assumption of cultural continuity, and its neglect of more dynamic processes of cultural transmission, interaction, and adaptation [2–4].

The historical development of the concept of culture in anthropology plays an important role in understanding how these frameworks emerged in archaeology. Tylor (1871) provided one of the earliest formal definitions of culture as “that complex whole which includes knowledge, belief, art, morals, law, customs, and any other capabilities acquired by man as a member of society” [5] This definition framed culture as a universal and holistic phenomenon, a view that endured well into the early 20th century (e.g., [6]). Later, culture was increasingly conceived as an abstraction (e.g., [7]), emphasizing its role as an analytical construct rather than a tangible entity, with boundaries and characteristics defined by researchers rather than intrinsic to human groups themselves. This shift that led to new definitions focused on symbolic and extrasomatic processes (e.g., [8]). Although these definitions played a major role in shaping anthropological thought, they were primarily developed for studying historically known societies, and their applicability to prehistoric archaeology has been widely debated [9,10].

Archaeologists have long grappled with how to apply cultural concepts to past human populations. Early 20th-century archaeology was heavily influenced by Gustaf Kossinna (1911) [11], who promoted a direct link between material culture and past ethnic groups—a view later criticized for its simplistic “one people, one pot” logic and its misuse for nationalist purposes [12,13]. Childe (1929, 1950) [14,15] attempted to refine this model by framing archaeological cultures as adaptive responses to environmental pressures, yet his approach still relied on the classification of material culture into discrete units. Phillips and Willey (1953) [16] challenged the assumption that archaeological cultures correspond to past social units, emphasizing instead that they are modern analytical constructs.

More recently, scholars have critiqued the culture-history paradigm for its failure to capture the fluidity of cultural transmission. Shennan (2002) [4] and Tostevin (2012) [17] argue that cultural evolution operates through mechanisms that are often decoupled from the spatial and temporal boundaries imposed by traditional typologies. Mesoudi and Whiten (2008) and Smolla and Akçay (2019) [18,19] highlight the role of transmission biases and network structures in shaping cultural change, which the culture-history approach overlooks. Reynolds & Riede (2019) [2] explicitly point out that many European Upper Paleolithic (EUP) technocomplexes are artifacts of archaeological classification rather than reflections of past human social realities. Similar critiques have been raised regarding the assumption that lithic industries can be treated as stable, coherent cultural markers [10,20].

In their work, Feinman & Neitzel (2020) [3] argue that culture-history approaches often reify archaeological constructs into fixed entities, neglecting social networks, collective action, and multiscalar interactions. Instead of treating cultures as discrete entities, they advocate for approaches that accommodate heterogeneous and overlapping social structures. This aligns with critiques of Upper Paleolithic cultural taxonomies, which are often defined based on lithic typologies but lack clear theoretical and empirical foundations [2].

Given these limitations, alternative methodologies have been proposed to analyze prehistoric cultural variability without relying on rigid classificatory schemes. Network analysis, for example, provides a means of visualizing and quantifying cultural transmission beyond typological constraints [3]. Similarly, eco-cultural niche modeling has been applied to study the relationship between environmental conditions and cultural adaptations [21,22]. The Cohesive Adaptive Systems (CAS) framework, proposed by d’Errico & Banks [23], integrates cultural and environmental data to provide a more dynamic understanding of culture-climate interaction. These perspectives allow archaeologists to move beyond rigid classifications and embrace the complexity of past social dynamics.

One of the most direct ways to investigate past social identities and interaction networks is through personal ornaments, which provide evidence of symbolic expression, social differentiation, and connectivity. Unlike functional artifacts such as stone tools, personal ornaments—beads, pendants, assorted in bracelets, necklaces or sewed on clothes—are designed to be displayed on the body and communicate a wide variety of meanings. The ethnographic record provides an ample repertoire of non-mutually exclusive functions that ornaments fulfil. They play a key role in the construction of individual identities [24]. They can signal gender and age [25], mark the social status of individuals, assume particular meaning in ritual and funerary contexts [26], reflect long-distance interactions and signal social networks [27], and indicate group identity and linguistic affiliations [28]. It has also been proposed that symbolic artefacts evolve at a faster pace than functional artefacts thus providing a supplementary proxy when investigating cultural change [29]. Given their wide geographic distribution, discrete typological variation, and abundance in the archaeological record, personal ornaments offer a robust dataset for statistical analyses of cultural networks. However, their performance in assessing cultural coherence and resilience remains an empirical question, to which this study contributes.

Although the typology and technology of personal ornaments have been widely studied [30–33], their variation in time and space have only been the subject of pioneering studies [34–36]. The earliest known personal ornaments date to approximately 140 thousand years ago (ka) [37–39] and consist of a small range of naturally and deliberately perforated marine shell species. It is only after around 45 ka, in the Eurasian continent, that personal ornaments made out of diverse raw material types (e.g., amber, antler, bone, fossils, ivory, jet, shells, stone, teeth etc.) begin to be found within the same archaeological assemblages [36,40–42].

A number of studies have aimed to utilise differences in personal ornaments to highlight distinct groups within prehistoric cultures [30,42–46]. Four studies concern two consecutive Palaeolithic technocomplexes: the Aurignacian and the Gravettian [42,43,47,48]. Vanhaeren and d’Errico (2006) [42] analysed a georeferenced database of Aurignacian personal ornaments and concluded that they mirrored the ethnolinguistic geography of Europe during the early stages of human settlement by modern humans. They identified fourteen sets of sites (fifteen including the Near East set) that were arranged in a cline which traversed counter-clockwise from the Northern Plains to the eastern Alps, passing through western and southern Europe and interpreted this result as reflecting ethnolinguistic variability. Another group of researchers, Kovacevic et al. (2015) [48], applied Approximate Bayesian Computation to the same dataset created by Vanhaeren and d’Errico (2006) [42]. By running two types of simulations, one in which group interactions were affected by cultural similarity and one in which it was not, they concluded that the personal ornaments from the Aurignacian could not be used to identify ethnic groups. No attempt was made in either of these studies to investigate whether Isolation-by-Distance (IBD) played a role in differences in personal ornaments found at sites. IBD is a mechanism in population genetics whereby individuals have a higher likelihood of gene exchange with conspecifics who are geographically closer to them [49,50]. Whether Aurignacian cultural groups can be identified based on differences in personal ornaments, and whether these differences can be explained by IBD, remain questions yet to be unraveled.

d’Errico and Vanhaeren (2015) [51] analysed grave goods (i.e., personal ornaments, tools, carved objects etc.) coming from Gravettian burials and found two, perhaps three, clusters of burials. These clusters, located in eastern Europe and western Europe, with a possible third in north-western Europe, each contained distinct sets of ornaments and other grave goods. The authors concluded that these clusters reflected cultural differences between distinct groups of people during the Gravettian. In a more recent work, Baker et al. (2024) [43] analysed a georeferenced dataset of personal ornaments found at Gravettian occupation and burial sites and demonstrated that nine cultural entities could be recognised within this technocomplex, and that the differences in personal ornament associations were not explainable by IBD alone. These entities were organised in an east-to-west cline along the length of Europe. Upon examining the results of a recent palaeogenomic study conducted by Posth et al. (2023) [52], it appears that a substantial degree of overlap existed between genetic and cultural diversity revealed by ornaments types but that other clusters identified by personal ornaments did not present genetic counterparts due to the lack of genetic data in some regions.

Thanks to Aurignacian and Gravettian personal ornament datasets, the opportunity to statistically compare the temporal and spatial variation of bead associations during the Initial Upper Palaeolithic is now well within our grasp. Therefore, in this study we combined and updated the previously created datasets of Vanhaeren and d’Errico (2006) [42], reviewing the new contexts published to date, and Baker et al. (2024) [43] which detail the personal ornaments found at layers and sites attributed to the Aurignacian and Gravettian cultures, respectively.

The main objective of this paper is to document personal ornament during the first half of the Upper Paleolithic (42–24 ka) as a case study to assess the role of symbolic traditions and regional connectivity. By integrating a broader analytical framework, we seek to move beyond the limitations of the culture-history paradigm and contribute to ongoing discussions on how prehistoric cultural patterns should be interpreted.

More specifically, the first aim of the present study is to document the degree of similarity in personal ornament use between archaeological contexts dated to between 42 ka and 25 ka and attributed to the Aurignacian sensu lato and the Gravettian to explore resilience in symbolic behaviour. Second, we seek to use the available Aurignacian bead dataset to evaluate whether observed differences in personal ornaments during the Aurignacian can be attributed to IBD or to cultural geography. Third, we aim to extend this comparison at a geographic level to establish whether patterns of continuity are documented in each region. We also wish to establish whether some bead-types are more informative than others to mark affiliation to a technocomplex. Our results identify strong continuity, including at regional levels, while highlighting that sites attributed to the Aurignacian displays geographically smaller clusters of sites sharing similar bead-types. We interpret this pattern as signaling an increase in cultural connectivity with the transition from the Aurignacian to the Gravettian. We will argue that the integration of personal ornaments in the analysis of Upper Palaeolithic cultural adaptations is essential to disentangle their complex nature, and identify patterns of continuity in their symbolic world and reassess the concept of archaeological technocomplex.

### Chronological and geographical context

Our study focuses on personal ornaments found in Europe during a time between *circa* 42–24 ka. This period is characterised by the presence of two wide-spread technocomplexes known as the Aurignacian (*circa* 42–32 ka cal. BP [53–55] and the Gravettian (*circa* 34–24 ka cal. BP [56–58]. During this time the European continent underwent eight stadial/interstadial cycles which resulted in rapidly fluctuating climatic conditions [59–64]. The Aurignacian is further subdivided into the Proto and Early Aurignacian [22,65,66] with the latter also having a small presence in the Levant [67–69]. The Gravettian is often described as having numerous regional variants, or ‘*facies*’ [70–76], and is found exclusively in Europe. Both technocomplexes possessed their own distinguishing features. Both the Aurignacian (*sensu lato*) and the Gravettian, along with their respective variants are characterised by distinct lithic and bone technologies as well as tool types [57,65,76–83]. Symbolic expression, exemplified by cave and mobiliary art, also differs between these two technocomplexes [84–97]. A supplementary difference between the two technocomplexes concern their funerary practices. Whilst the Gravettian boasts an abundance of elaborately buried individuals across its geographic range, there is a marked dearth of burials, elaborate or otherwise, throughout the Aurignacian [47,98–101]. Thus, despite the extensive time periods during which these two technocomplexes existed, the distinct diagnostic traits detailed above facilitate the precise identification of either one or the other within the archaeological record. This therefore allows, in well-preserved sites, personal ornaments to be reliably attributed to one of the two technocomplexes.

## Materials and methods

**Fig 1:**
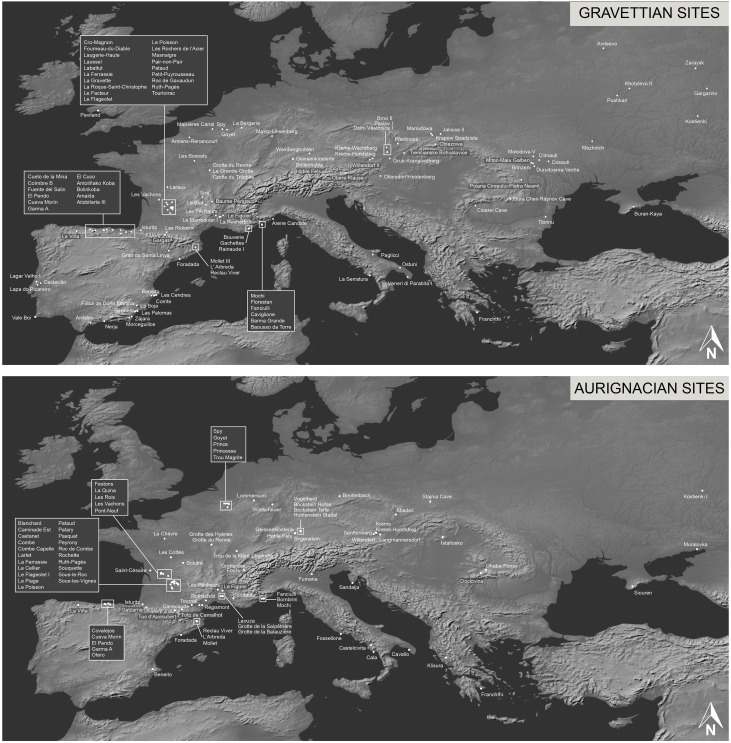
Maps of the Aurignacian and Gravettian sites included in the analysed datasets. Maps created using the Free and Open Source QGIS. Background maps (1:10m Gray Earth) used from Natural Earth. Free vector and raster map data @ naturalearthdata.com.

Our dataset is comprised of updated versions of four previously published databases assembling information on personal ornaments from the Aurignacian [42] and the Gravettian burial and occupation sites [43,51,102,103] (Fig 1; S1 Dataset Worksheets ‘B’, ‘C’, ‘D’, and ‘E’). Following the publication of Baker et al. 2024, the research team was expanded to improve the reliability of cultural attributions, addressing potential ambiguities in site classification. This included a more extensive review of literature in original languages, stricter criteria for associating ornaments with a specific technocomplex, and an increased emphasis on radiocarbon dating when available. As a result, the updated Gravettian dataset includes: the addition of 3 burials and 30 occupation sites, the removal of 9 burials and 8 occupation sites, the addition of radiocarbon dates for 2 burials and 4 occupation sites, the removal of radiocarbon dates for 1 burial and 2 occupation sites, and the addition of 31 ornament types. For the Aurignacian, 4 occupation sites were added.

The unified dataset only includes sites whose cultural attribution is unequivocal, either based on diagnostic artifacts, well-established archaeological literature, or consistent radiocarbon dates. While sites without radiocarbon dates have been included, priority was given to those with secure chronological frameworks, and whenever possible, attributions were cross-validated through independent lines of evidence. Some areas and periods presented greater challenges in attribution due to gaps in the archaeological record or discrepancies in site reporting. We excluded sites attributed to the Levantine Aurignacian [67,68,104] from our analyses since the Gravettian is not documented in the Near East. Sites were attributed to specific groups following the cultural geography identified by two previous studies [42,43] based on similarity in bead-types associations and geographic proximity.

We created mutually exclusive bead types, taking into account cross-cultural studies on the classification of beads [33,105–107] and on criteria used to classify archaeological artefacts [107–109]. Ornament types were first categorized by raw material. For shell ornaments, the genus and species were considered. When multiple species of a genus were indistinguishable to the naked eye, we only recorded the genus. Shell species taxonomy has undergone significant development since the earliest papers detailing Palaeolithic ornaments were published [33,110,111]. Care was therefore taken to ensure that species in older literature were updated to modern nomenclature. To prevent attribution to multiple taxa when referring to the same shell species, we used the World Register of Marine Species (WoRMS; https://www.marinespecies.org) to characterize the species [112]. For animal teeth, the genus, species and anatomical attribution were taken into account. For fully shaped ornaments we created mutually exclusive types based on five main criteria: (1) raw material (e.g., amber, bone, antler, ivory, stone), (2) general outline, particularly as the ornament would appear when worn and viewed by others (e.g., round vs. elongated), (3) volume morphology (e.g., spherical, ellipsoidal, flat), (4) mode of attachment (e.g., single or multiple perforations, grooves). When objects exhibited transitional features between categories, we prioritized distinct morphological traits and attachment methods to ensure clear classification. However, we acknowledge that some shaped ornaments presented challenges due to gradual stylistic transitions. These cases were carefully reviewed, and any uncertainties were minimized through comparative analysis of similar artifacts from well-dated contexts. A small number of perforated bones, described in the literature as ornaments, display features typical of bone fragments regurgitated by carnivores [113]. These specimens were excluded from the dataset. One limitation of our study is that we did not directly examine all the personal ornaments included in our dataset. Some information was obtained from published sources, and in these cases, we carefully reviewed photographs and descriptions to verify the identification. For shell ornaments, when species-level identification was uncertain, we recorded them as sp. to avoid misclassification. Another limitation concerns the uncertainty regarding the origin of shells—whether they are of marine (i.e., from the shores available to Paleolithic people at the time) or fossil origin. This is particularly relevant for *Glycymeris* sp. and *Antalis* sp., especially those found at sites far from the coast, where the use of fossil specimens is more likely. Determining the origin with certainty would require in a number of cases direct study of the specimens in future research.

### Statistical analyses

We employed a Jaccard distance index to construct distance matrices for personal ornament dissimilarity between sites [29,114,115] (S2 File Worksheets ‘A’ and ‘C’). This index is well-suited for archaeological studies because it ignores absence data [116,117]. With the exception of the Mantel tests (see below), sites presenting only unique ornament types were excluded from the distance matrix [118].

To assess whether geographic distance played a role in determining the differences in ornament type associations we used a Mantel test [119]. We used Jaccard and Euclidean distances to construct the cultural and geographic dissimilarity matrices, respectively (S2 File Worksheets ‘B’ and ‘D’). This was performed on two datasets, one containing Aurignacian sites and the other Gravettian sites. We also produced Mantel correlograms [120,121] for both datasets to ascertain the degree of spatial autocorrelation, which measures the higher than average similarity between sites for a given distance. We used three distance classes for each dataset to account for patterns observable at different scales [122] – these were 500, 250 and 100 km. Whilst the Mantel test correlation examines the overall relationship between two distance matrices, the Mantel correlogram delves deeper into its internal organisation.

We performed a Principal Coordinates Analysis (PCoA) on the cultural distance matrices using PAST software. A PCoA was chosen due to its ability to handle binary data [123]. A PCoA was performed both on the Aurignacian and Gravettian sites at a European and regional scales.

In order to assess to what degree the technocomplex to which a site belonged explained the observed differences in the bead-type variance, we utilised a perMANOVA [124,125] using the ‘Adonis2’ package in R [125]. A perMANOVA is a type of ANOVA, which investigates the degree of variance in the centroid position and dispersion of different groups (here technocomplexes).

We performed k-means clustering analysis to identify clusters of sites based on the personal ornaments found at different sites with the ‘Silhouette’ method [126–128] using the ‘factoextra’ package in R [129]. K-means clustering works by organising a dataset into distinct groups (clusters) based on the similarity of data points (sites) and their respective variables (personal ornament types) with no *a priori* knowledge of group affiliation, with each cluster represented by its centroid [130,131].

To statistically asses difference in the number of ornament types found at sites of different functions (*i.e.*, Aurignacian occupation, Gravettian occupation, Gravettian burial), we applied a Kolmogorov-Smirnov test [132,133] using the ‘tidyverse’ package [134] in R. When comparing multiple groups, this test evaluates how closely their sample distributions align by measuring the maximum vertical difference (D) between their cumulative distribution functions. This provides insight into the similarity of the distributions. To examine differences in shaped ornament types between the Aurignacian and Gravettian, we performed a seriation using the algorithm described by Brower and Kile (1988) [135]. This method reorganizes the presence-absence data matrix to concentrate occurrences along the diagonal, enhancing pattern recognition.

### Archaeological Similarity Networks

Network analysis has become an increasingly valuable tool in archaeology for exploring patterns of interaction, connectivity, and cultural transmission among prehistoric communities [136]. Traditional typological and spatial approaches often assume static boundaries between archaeological entities, whereas network-based methods provide a more dynamic perspective on how material culture circulates across regions. By representing sites as nodes and their degrees of similarity as edges, network analysis allows researchers to quantitatively assess relationships between archaeological assemblages, identify clusters of interaction, and detect potential pathways of information exchange. This approach is particularly useful for evaluating the extent of cultural cohesion or divergence within and between technocomplexes, as well as for testing hypotheses related to mobility, contact networks, and regional adaptation.

We employed Archaeological Similarity Networks (ASN) to investigate the interconnectedness among various archaeological sites by assessing their degrees of similarity based on the Jaccard similarity index [114]. In the context of ASN, individual nodes correspond to distinct archaeological sites, while edges connecting nodes indicate the extent of similarity between two sites, with the weight (thickness) of these edges directly reflecting the similarity value [137–139]. A higher level of similarity between two archaeological sites is represented by a thicker edge, whereas a thinner edge signifies a lower similarity between the two nodes. To improve readability and highlight meaningful connections, we applied a threshold limit of 0.2, meaning that only site pairs with a similarity value above this threshold were plotted in the graph. Various statistical measures can be used to assess network connectivity and structure. For each computed network, we calculated network density and interval statistics. Additionally, for each individual technocomplex ASN, we calculated the Similarity Radius, as used in [44]. A more detailed description of ASN construction and analysis can be found in Pereira et al. (2023) [44]. The R code used for this study was adapted from the same article.

## Results

Hunter-gatherers living in Europe between 42 ka and 25 ka used 215 discrete types of ornaments, found at 202 sites (S1 Dataset Worksheets ‘A’, ‘D’ and ‘E’). These are composed of 148 types at 98 Aurignacian sites and 141 types at 132 Gravettian sites. A total of 105 ornament types were fashioned out of shells, and 28 were made from mammal teeth. Among these, 15 types came from herbivores, omnivore, and rodent species, including beaver, elk, fallow deer, hare, horse, ibex, red deer, reindeer, steppe bison and wild boar. Another 13 types were derived from carnivores, including badger, bear, fox, hyena, lion, lynx, and wolf. Human teeth were also used as ornaments. Two types were fashioned out of fish teeth (gilt-head bream and shark). Seventy-nine were formed from other raw materials. Forty-four types were crafted from ivory, 19 from bone and 7 from antler. A further 5 types were made from stone, and four from fish vertebrae, amber, a modified tooth root and jet.

**Fig 2:**
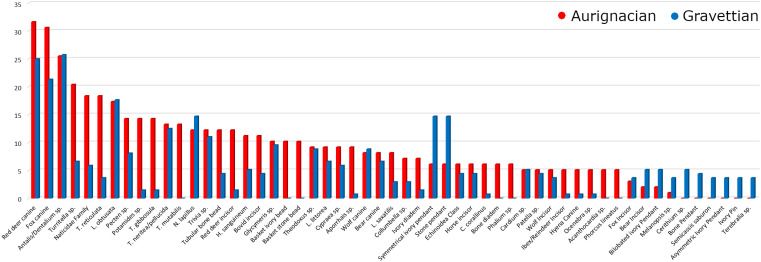
Percentage of Aurignacian and Gravettian sites at which the most common personal ornament types were found (defined as personal ornaments that appear in 5 or more sites).

Just over a third (34.42%) of the ornament types are common to the two technocomplexes. Among the ornament types found at five or more sites, 76.36% are present in both (Fig 2). The Aurignacian was characterised by fewer ornament types made of shells (Aurignacian N = 67, Gravettian N = 78), more types made of teeth (Aurignacian N = 26, Gravettian N = 24) and other raw materials (Aurignacian N = 55, Gravettian N = 40). Concerning types made of teeth, the Aurignacian had fewer types of mammal carnivore species (Aurignacian N = 11, Gravettian N = 12) and more types of mammal herbivore species (Aurignacian N = 13, Gravettian N = 10), whilst both had 1 type from a fish species. The Aurignacian is richer in ornaments made from a variety of raw material, *i.e.*, ivory (Aurignacian N = 31, Gravettian N = 24), antler (Aurignacian N = 7, Gravettian N = 0), stone (Aurignacian N = 4, Gravettian N = 2) except jet (Aurignacian N = 0, Gravettian N = 1) and the same number of types of bone (Aurignacian N = 11, Gravettian N = 11).

The top 5 most frequent ornaments types associated with both technocomplexes are, in decreasing order, fox canines, red deer canines, *Antalis*/*Dentalium* sp., *Turritella* sp. and Naticidae Family shells (Fig 2). The top 5 most common ornament types during the Aurignacian are red deer canines, fox canines, *Antalis*/*Dentalium* sp., *Turritella* sp., Naticidae Family and *Tritia reticulata* shells (final two both with 18.37%) whilst the five most common ornament types during the Gravettian are *Antalis*/*Dentalium* sp. shells, red deer canines, fox canines, *L. obtusata* shells, *N. lapillus*, symmetrical ivory pendant and stone pendant (final three all with 14.7%).

Among the animal tooth ornament types, red deer incisors and Bovid incisors, commonly found in the Aurignacian, are rare in the Gravettian. The same applies to ornaments made out of certain shell species (*i.e.*, Naticidae Family, *Potamides* sp., *Tritia gibbosula*, *Apporhais* sp. and *Clanculus corallinus*) which were widely worn during the Aurignacian but only sparsely in the Gravettian. Ivory diadems are found at numerous Aurignacian sites but only at two Gravettian sites. In contrast, symmetrical ivory pendants, widespread in the Gravettian, were infrequent in the Aurignacian. Widespread ornament types worn in the Aurignacian, such as *Phallium* sp., bone diadems and basket-shaped beads fashioned out of ivory and stone, have so far not been found at Gravettian sites.

**Fig 3:**
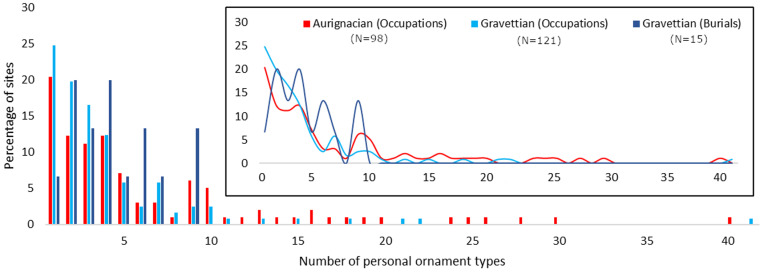
Number of ornament types found at Aurignacian and Gravettian occupations and primary burial sites.

On average, more personal ornament types are found at Aurignacian (N = 7) than at Gravettian occupation sites (N = 4) and at Gravettian primary burial sites (N = 4). The sites appear to be divided in two groups regarding the number bead-types present, with one group composed of sites which are characterized by a higher number of bead-types present (N > 10) and another group which is characterized by sites with fewer bead-types present (N ≤ 10) (Fig 3). The distributions between the Aurignacian occupations and both the Gravettian occupations and burials are significantly different (Table 1 & Supplementary Results A in S1 File). No such difference is observed between the Gravettian burials and Gravettian occupations (Table 1 & Supplementary Results A in S1 File). The results when the individual human remains are treated as single units of analysis are shown in S1 File.

**Table 1:** Results of the Kolmogorov-Smirnov test calculated for the bead-type associations recorded at Aurignacian occupation and Gravettian occupation and burial sites.

	D Value	P-value
Aurignacian and Gravettian occupations	0.18848	0.0132
Aurignacian occupations and Gravettian burials	0.26777	0.0346
Gravettian occupations and Gravettian burials	0.11285	0.6376

### Testing Isolation by Distance as a cause of ornament-type diversity

The application of the Mantel test [119] to the Aurignacian dataset reveals a non-significant, near-zero correlation between geographic and cultural distances based on the personal ornament variability (Table 2). The application of the Mantel test to the Gravettian dataset shows a significant, but very weak correlation between geographic and cultural distances (Table 2).

**Table 2:** Results of the Mantel tests calculated for the bead-type associations recorded at Aurignacian and Gravettian sites.

Dataset	R	P-value
Aurignacian	+0.03997	0.10589
Gravettian	+0.05417	0.001998

**Fig 4:**
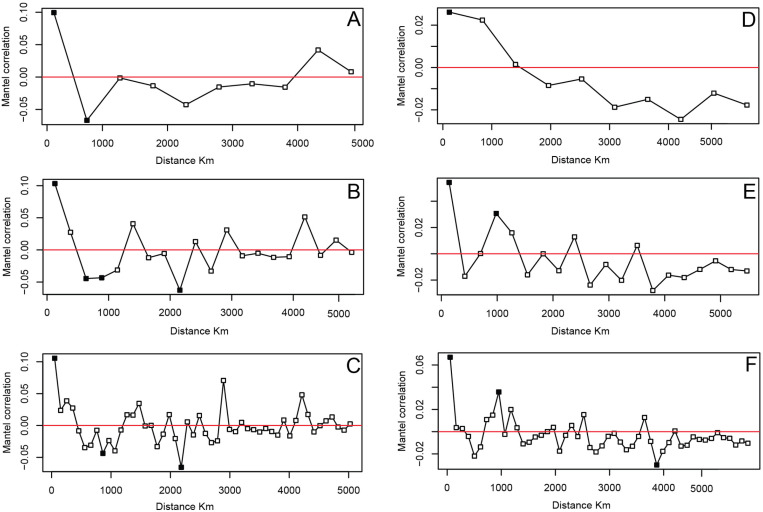
Mantel correlograms established for the ornament-type associations recorded at Aurignacian (a, b, c) and Gravettian sites (d, e, f). Units of geographic distance are 500 km (a, d), 250 km (b, e), 100 km (c, f). Black squares indicate significant P-values, white squares non-significant P-values.

The Aurignacian Mantel correlograms display some significant positive autocorrelation up to geographic distances of approximately 300 km and significant negative autocorrelation at distances greater than approximately 800 km (Fig 4a–4c). The Gravettian Mantel correlograms display significant positive autocorrelation up to geographic distances of approximately 1000 km and significant negative autocorrelation at distances over 3800 km (Fig 4d–4f).

### Parametric Coordinate Analysis

**Fig 5:**
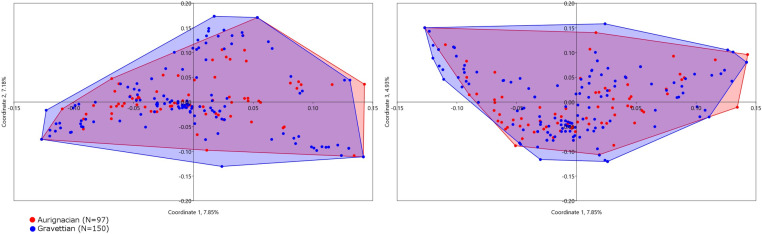
First, second and third axes of the Principal Coordinate Analysis of the bead-type association recorded at Aurignacian and Gravettian sites.

**Fig 6:**
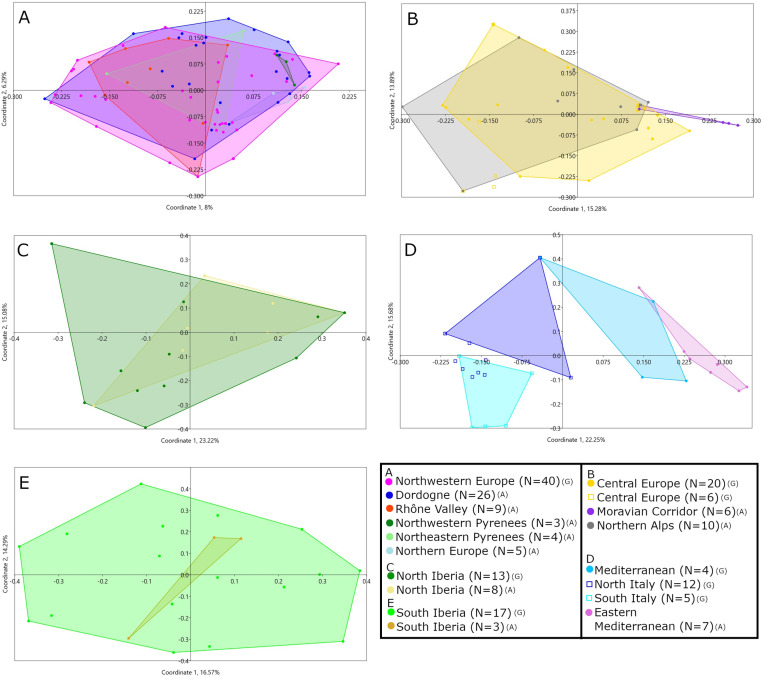
First and second axes of the Principal Coordinate Analysis of bead-type association recorded at Aurignacian and Gravettian sites in a) North-western Europe, b) central Europe, c) north Iberia, d) western Mediterranean and e) south Iberia. Circle = occupation, square = burial. (A) = Aurignacian, (G) = Gravettian.

The first, second and third axes of the Principal Coordinate Analysis (PCoA) [123] of the Aurignacian and Gravettian datasets reveals an almost complete overlap of the bead-type associations found in the two technocomplexes (Fig 5). At a regional scale, using the groups identified in Vanhaeren and d’Errico (2006) [42] and Baker et al. 2024 [43] for the Aurignacian and the Gravettian, respectively, we observe a partial overlap between bead-type associations (Fig 6). However, some of these overlaps are due to bead-type associations found at single sites (Fig 6d). For the first and third axes of each of these PCoAs and for the North and South Iberia groups combined, see Supplementary Results B in S1 File.

### Permutational Multivariate Analysis of Variance

The Permutational Multivariate Analysis of Variance (perMANOVA) of the Aurignacian and Gravettian datasets yielded a statistically significant but low R^2^ value of 0.00968 (p = 0.001) with an F-model of 2.4048 (Table 3), indicating that the technocomplex to which bead associations belong accounts for only 1% of the observed variance. However, a much higher value is observed when perMANOVA is applied only to fully-shaped ornaments (R^2 ^= 0.03575 at a p-value = 0.001, with an F-model of 4.0785). In contrast, the non-shaped personal ornaments, (*e.g.*, teeth, shells*)*, explain far less of the variation (R^2 ^= 0.00939 at a p-value = 0.007, with an F-model of 2.0957).

**Table 3:** Results of the perMANOVA of the combined Aurignacian and Gravettian excluding sites only presenting bead-types not shared with any other site.

Dataset	R^2^	P-value	F-model
Aurignacian and Gravettian ~ Technocomplex	0.00968	0.001	2.4048
Aurignacian and Gravettian ~ Technocomplex (shaped ornaments)	0.03575	0.001	4.0785
Aurignacian and Gravettian ~ Technocomplex (non-shaped ornaments)	0.00939	0.007	2.0957

### Seriation

**Fig 7:**
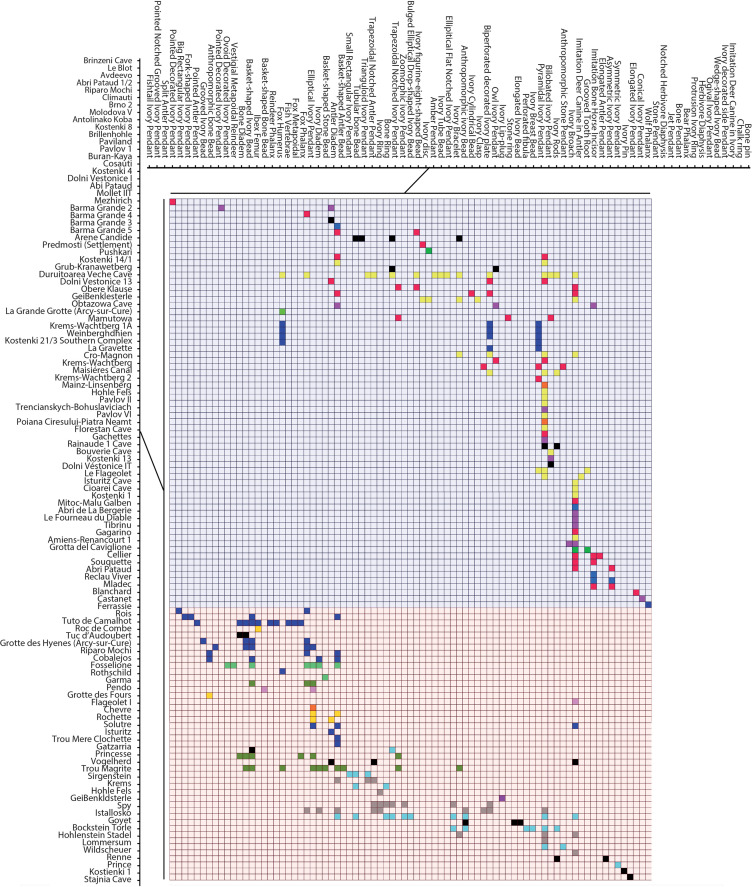
Seriation analysis of the bead database for shaped personal ornaments from Aurignacian and Gravettian sites. Squares indicate the occurrence of ornament types at sites. The colour of the square represents the geographical set to which the site belongs. Shaded background colours represent the technocomplex to which the sites belong, Red = Aurignacian, Blue = Gravettian.

Since shaped ornaments account for over three times the variation in the data compared to both all personal ornaments combined and only the non-shaped personal ornaments, we conducted a seriation analysis to explore this pattern further. The seriation of the Aurignacian and Gravettian shaped personal ornaments found at sites from different European regions reveals two distinct patterns (Fig 7). The Aurignacian is characterised by 37.5% more types of shaped personal ornaments than the Gravettian (N = 55 and N = 40, respectively) which are more varied in terms of raw material and shape than the latter. It also appears that the Aurignacian is geographically structured around a north-south cline. Within this cline, two main sub-groups are observed, one encompassing sites located at the north of the Alps and along Atlantic Europe and the other including sites from the Mediterranean region. The Gravettian presents fewer shaped personal ornament types mostly represented by symmetric drop-shaped ivory pendants, stone pendants and tubular bone beads. Contrary to the pattern observed in the Aurignacian, these types are found at sites from different regions of Europe, thus not revealing any clear geographic structure.

### K-means clustering algorithm

**Fig 8:**
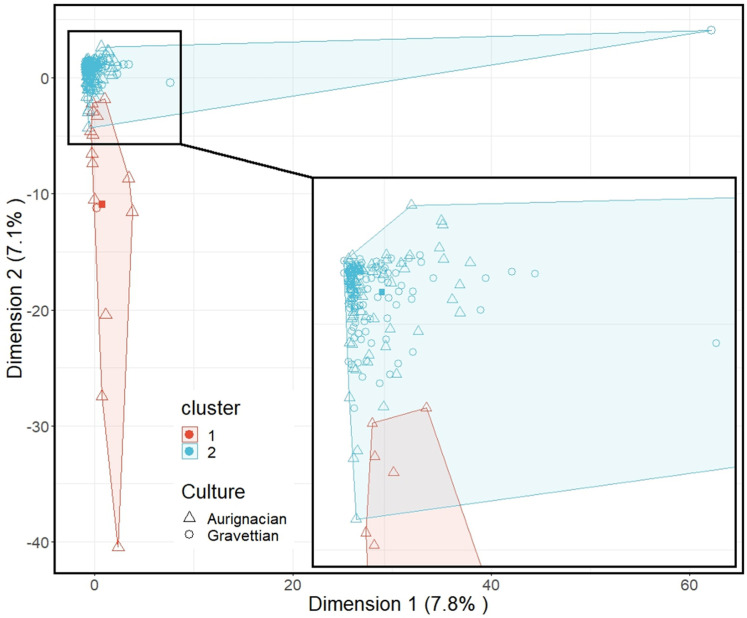
K-means clustering analysis of the bead-type association recorded at Aurignacian and Gravettian sites. Square = cluster centroid. Red = Cluster 1, Blue = Cluster 2.

The k-means clustering algorithm [128,130] groups the sites into two clusters (Fig 8). The smaller cluster (red) (N = 15) is formed by Aurignacian sites (N = 14) from the Dordogne, the Rhône Valley, the eastern Pyrenees and Italy in addition to a single Gravettian occupation site (Riparo Mochi) from the Ligurian coast. The larger cluster (blue) (N = 232) is formed by Aurignacian (N = 83) and Gravettian (N = 149) sites from across the entirety of Europe. The smaller cluster is characterised by sites yielding numerous types of ornaments (N > 10) which are principally made of marine shells. The majority of these shells were either available from both Atlantic and Mediterranean shores or only from the latter. The larger cluster is characterised by sites yielding fewer ornament types (N ≤ 10) which tend to be made of teeth, Atlantic and Mediterranean shells, or were shaped from other raw materials. The differences between the two clusters echo the observed variation in the distribution of the number of types per site (Fig 3).

### Archaeological Similarity Networks

**Fig 9:**
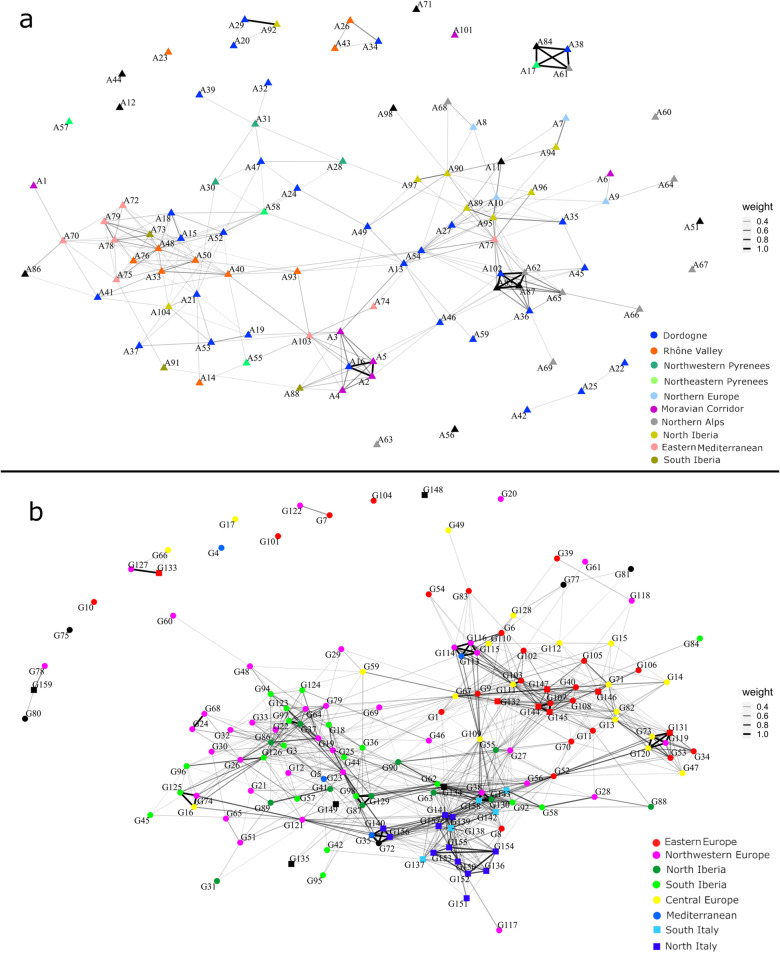
Archaeological Similarity Network plotted with Fruchterman-Reingold layout for, a) Aurignacian and, b) Gravettian full dataset. The ASNs’ edge weight (thickness) caption to the right of the corresponding network corresponds to the pairwise similarity value between connected nodes. Symbol code: circle – Occupation; square – Burial.

The Aurignacian and the Gravettian networks differ in their degree of network density with the latter being more densely connected (0.0544919 and 0.07615213, respectively; Fig 8 & in Supplementary Results C in S1 File). The Aurignacian network (Fig 9a) comprises three clusters (group of highly interconnected nodes) of sites, two of which show a core area with very high interconnectivity. One cluster (right) is principally composed of sites from the Dordogne, north Iberia and north of the Alps, a second (left) of sites from the Rhône valley, the Dordogne and the eastern Mediterranean, and a third (centre bottom) smaller group mostly including sites from the Moravian corridor. The Gravettian network is also composed of three clusters (Fig 9b). The largest cluster, on the left of the graph, contains sites from north-western Europe and the Iberian Peninsula. Another cluster (centre bottom) is principally composed of burials from Italy, with the inclusion of some occupation sites from north-western Europe. The cluster on the right is composed of sites from eastern and central Europe. Overall, both networks show an east-west cline, though this is far more visible in the Gravettian network.

**Fig 10:**
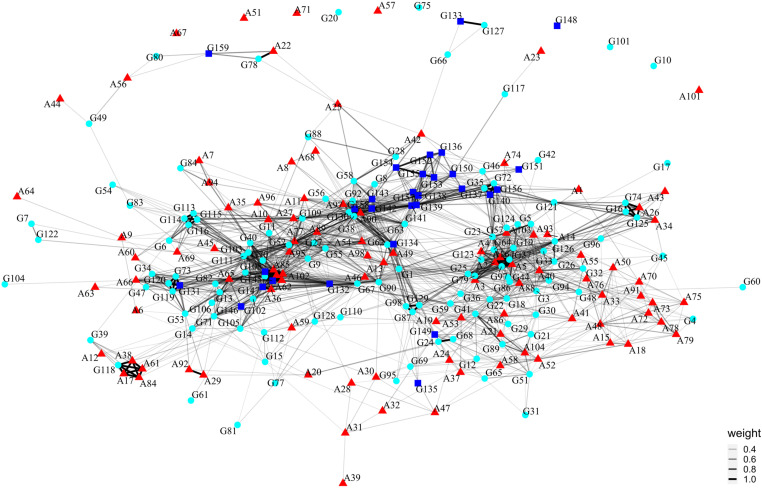
Archaeological Similarity Network for Aurignacian and Gravettian full dataset plotted with Fruchterman-Reingold layout. The ASNs’ edge weight (thickness) caption to the right of the corresponding network corresponds to the pairwise similarity value between connected nodes. Colour and symbol code: red triangle – Aurignacian; blue square – Gravettian Burials; cyan circle – Gravettian Occupations.

In the combined network there are tentatively three clusters of sites (Fig 10). One, at the right, and another, on the left, have sites from both the Aurignacian and the Gravettian in which we see a high number of links between sites regardless of the technocomplex. Another group, at the centre-top of the graph, is composed primarily of Gravettian burials from the Italian Peninsula, in addition to some Aurignacian and Gravettian occupation sites. The majority of the Aurignacian and Gravettian sites present links with other sites regardless of their cultural attribution. The combined network encompasses 248 nodes connected by 1817 edges and has a density of 0.0593248 (Supplementary Results C in S1 File).

The maximum and average geographical distances between Gravettian sites (5486 km and 1504 km, respectively) connected in the network were greater than between Aurignacian sites (4717 km and 988 km, respectively) (Supplementary Results C in S1 File).

## Discussion

This study represents the first attempt to explore variations in bead-type associations quantitatively within the earliest two main Upper Palaeolithic cultural phases. Our results reveal previously unreported major similarities in the personal ornament types worn during the Aurignacian and Gravettian, with just over 1% of the bead types at a given site explained by their cultural attribution. These stark similarities are observed at both continental and regional scales. This suggests that, despite clear differences in certain technological and symbolic domains, Aurignacian and Gravettian populations shared similar preferences in the choice of items used to culturalise their bodies. This continuity is further confirmed by the combined network analysis (Fig 10), which highlights strong links between personal ornament types across the two technocomplexes. Our findings challenge the notion that Aurignacian and Gravettian technocomplexes represent monolithic cultural entities, instead supporting the view that they reflect adaptive cultural processes with varying degrees of continuity in ornamentation practices. More broadly, this underscores the importance of approaching Upper Paleolithic cultural change by independently examining different domains of material culture and developing methods to identify evolutionary patterns. Continuity in personal ornament types, however, does not necessarily imply that Aurignacian and Gravettian populations arranged or interpreted these ornaments in the same way. Ethnographic data indicate that similar bead types can be arranged differently to signal diverse social and group identities, including ethnic and social affiliations [25,106]. Crucially, our study does not assume that ethnic groups existed in the Upper Paleolithic in the way they are understood in production or state societies. Instead, we acknowledge that identity signaling through ornaments likely reflected multiple dimensions, including regional traditions, social roles, and individual expression, which may or may not correspond to broader cultural groupings. An analysis of bead-type associations alone cannot determine whether the arrangement of beads on the body changed between these two technocomplexes. One possible approach to inferring such changes is by examining the quantity of the same bead type in Aurignacian and Gravettian layers—under the assumption that large variations in proportions could indicate different ways of wearing them. However, this type of information is only available for a limited number of sites where systematic fine-mesh sieving was conducted, a condition that varies among older excavations. Evidence that significant variations in bead proportions may reflect different ways of wearing ornaments comes from Franchthi Cave (Greece), where a fully recovered shell bead assemblage provides clues about differences in ornament arrangement over time [140,141]. Further insights could come from detailed use-wear and perforation analyses, distinguishing between beads strung freely in necklaces and bracelets and beads sewn onto garments. Although important studies have focused on selected bead assemblages, this information is not yet available at a European scale, making it difficult to track changes in ornament arrangement between technocomplexes. Despite these limitations, we do know that ornament arrangement played a meaningful role in social differentiation during the Gravettian, thanks to numerous burials that provide direct evidence of bead placement on the body [101,142]. Several Gravettian burials show that individuals in the same region often used similar bead types but arranged them differently, suggesting that ornament arrangement contributed to signalling individual and social differences [143–145]. Although similar practices likely existed during the Aurignacian, the absence of primary burials from this period limits direct investigation. Future studies combining ornament typology, use-wear analysis, and contextual data from burials may help clarify how personal ornaments functioned as flexible markers of identity rather than fixed indicators of distinct ethnic groups.

An alternative way to assess the significance of the observed ornament continuity is to investigate whether it characterises all bead-type categories or some of them expressed cultural change better than others. We observed that shaped personal ornaments are those that varied by far the most between the Aurignacian and the Gravettian. Apparently, fully carved ornament types better marked the cultural divide between these two technocomplexes than those produced from minimally modified natural forms.

One may argue that cultural drift more heavily affects fully-shaped personal ornaments because completely carving a raw material gives more freedom in design than relying on natural morphologies. If this was the case, we would expect to identify a similar pattern in the future when analysing personal ornament variation throughout the Upper Palaeolithic. Another non-mutually exclusive explanation is that fully-shaped ornaments were preferentially produced and worn by individuals with special social roles and, for that reason, less submitted to rules applying to the other members of the group. This explanation is supported by the fact that apart from a few highly morphologically homogeneous types, such as Aurignacian basket beads and the Gravettian drop-shaped ivory beads [146], the vast majority of shaped ornaments feature high morphological variability. This greater variability suggests they were more susceptible to stylistic divergence, whether due to individual expression, social status, or cultural drift. If cultural change was expressed through personal ornaments, then shaped ornaments associated with social status may have been the most sensitive markers of such change.

Another aspect of our results worth discussing is the presence in the Aurignacian of sites featuring a significantly greater number of bead-types than at Gravettian occupation sites. This striking difference cannot be attributed to the number of bead-types used throughout the two technocomplexes, which is almost the same. Two hypotheses could explain this difference: the presence in the Aurignacian of more intensively frequented aggregation sites collecting a broader range of bead-types used by the groups living within a given region or that these sites may have captured the greater social complexity characterising Aurignacian societies compared to those of the Gravettian. This hypothesis is consistent with the observation that a remarkable similarity is observed in the number of bead-types found at Gravettian burial and occupation sites. In other words, single or multiple burials apparently collect the full array of beads used by a Gravettian community. Our analyses revealed an insignificant correlation between geographic and cultural distances during the Aurignacian and a low but statistically significant correlation during the Gravettian. A closer examination of the correlations at specific geographic distances reveals that the Aurignacian yields statistically significant values at certain geographic distances, however. Whilst the Aurignacian dataset presents significant positive autocorrelation at short geographic distances (300 km), the Gravettian dataset presents significant positive autocorrelation at much greater geographic distances (1000 km). The Gravettian was therefore organized into cultural zones that were over three times larger in diameter than those of the Aurignacian. This pattern suggests a shift in how cultural information was transmitted and maintained over space. The significantly wider zones of similarity in the Gravettian, coupled with the greater geographic distances between connected sites, suggest that Gravettian populations were part of larger, more extensively connected networks, allowing for the wider sharing of symbolic traditions related to body adornment. This finding aligns with previous archaeological evidence indicating increased long-distance lithic transport in the Gravettian, a higher density of sites, and greater mobility of materials used for symbolic purposes, such as ivory and marine shells. Additionally, the stronger east-west cline observed in Gravettian ornaments may reflect a long-term process of cultural homogenization following the initial dispersal of modern humans into Europe. In contrast, the Aurignacian ornament distribution, with significant cultural autocorrelation at short distances, suggests that early Upper Paleolithic social networks were more regionally structured, potentially due to a combination of founder effects, local adaptations, and smaller-scale interaction networks.

Network analysis of bead-type associations demonstrates that Aurignacian sites presented fewer connections than Gravettian sites. The interval statistics associated with the network analysis, specifically the Similarity Radius result, additionally confirm the greater geographic distances between connected Gravettian sites within the networks, as highlighted by the Mantel correlograms (Supplementary Results C in S1 File). A reasonable explanation for the difference between the two technocomplexes is that, as computer simulations suggest [147–149], populations that are moderately connected tend to conserve a higher level of material culture variability than those more highly connected. In highly connected networks, a strong degree of homophily can lead to greater conservatism, reinforcing existing traditions and limiting the incorporation of novel forms. This aligns with what we know about the Gravettian in other symbolic and technological behavioural domains [87,89,94,96,150,151]. The combined network analysis (Fig 10) further confirms that a high degree of continuity in personal ornament types existed between the two technocomplexes.

The stronger east-west cline observed in the Gravettian may be explained as the long-term outcome of the process of cultural homogenization following the first widespread arrival of modern human populations. Aurignacian bead geography would have been influenced by a founder effect gradually smoothed out during the Aurignacian and subsequent Gravettian.

## Conclusion

It has been argued based on more traditional archaeological proxies, such as lithic [152–154], osseous technology [155,156] and settlement patterns [150,157], that the Gravettian represents a novel cultural adaptation compared to the Aurignacian. We have highlighted, however, that a large continuity existed in the objects worn by the members of the two technocomplexes to adorn themselves. This continuity illustrates the complex nature of Upper Palaeolithic cultures. Our results indicate that they were characterised by asynchronous changes in different behavioural domains. Personal ornaments are instrumental in documenting at which pace these changes occurred and understanding how behavioural domains coevolved.

Differences between the Aurignacian and the Gravettian have nevertheless emerged in certain bead-types and in the spatial distribution of bead-type associations. The more elaborate among the fully-shaped ornaments, probably reflecting special social roles, are apparently those evolving at a higher pace and on which future research should focus when using personal ornaments to assess cultural evolution. The identification of larger regions sharing similar bead-types in the Gravettian compared to the Aurignacian clearly reflects the former was characterised by social networks extending over larger regions, possibly indicating increased population size in the Gravettian. Extending these analyses to the remainder of the Upper Palaeolithic could identify long-term trends or pulses in social networks possibly correlated with climate changes and population dynamics.

## Supporting information

S1 Dataset Archaeological site dataset. Database of the archaeological sites, layers, variables, and radiocarbon dates used in the analysis. (A) Worksheet containing each of the mutually exclusive bead types and their respective codes. (B) Worksheet containing dataset with only the Aurignacian sites. (C) Worksheet containing dataset with only the Gravettian sites. (D) Worksheet containing the personal ornaments and attributes of the Aurignacian sites. (E) Worksheet containing the personal ornaments and attributes of the Gravettian sites.
(XLSX)

S1 File Method and results. Extra supporting analysis.
(DOCX)

S2 File Results. Cultural similarity, geographical and chronological distance matrices. (A) Worksheet containing cultural distance matrix generated from the Aurignacian dataset. (B) Worksheet containing geographical distance matrix generated from the Aurignacian dataset. (C) Worksheet containing cultural distance matrix generated from the Gravettian dataset. (D) Worksheet containing geographical distance matrix generated from the Gravettian dataset.
(XLSX)
