# Supplementary material for: Multivariate analyses of Aurignacian and Gravettian personal ornaments support cultural continuity in the Early Upper Palaeolithic
Source: PLoS One. 2025 Jun 4;20(6):e0323148. doi: 10.1371/journal.pone.0323148 (PMC12136358; doi:10.1371/journal.pone.0323148)
Supplement: S1 File — Extra supporting analysis. (DOCX) [file pone.0323148.s002.docx]

Multivariate analyses of Aurignacian and Gravettian personal ornaments support cultural continuity in the Early Upper Palaeolithic

Supplementary Information

Francesco d’Errico^1,2^, Jack Baker^1^, Daniel Pereira^1,3^, Esteban Álvarez-Fernández^4^, Martina Lázničková-Galetová^5,6^, Solange Rigaud^1^

1. CNRS UMR 5199 De la Préhistoire à l’Actuel : Culture, Environnement, et Anthropologie (PACEA), Université Bordeaux, Talence, France

2. SFF Centre for Early Sapiens Behaviour (SapienCE), University of Bergen, Bergen, Norway

3. BonesLab, Dipartimento di Beni Culturali, Università di Bologna, Ravenna, Italy

4. GIR Prehusal, Dpto. de Prehistoria, Historia Antigua y Arqueología. Facultad de Geografía e Historia. Universidad de Salamanca. Calle Cerrada de Serranos s/n. E-37002 Salamanca

5. Moravian Museum, Zelný trh 6, 659 37 Brno, Czech Republic

6. UMR 7194 HNHP, Equipe NOMADE, CNRS / Muséum national d'histoire naturelle (MNHN) / UPVD Institut de Paléontologie Humaine, 1 rue René Panhard, 75013 Paris, France

**Corresponding author:**

Francesco d’Errico

CNRS UMR 5199 De la Préhistoire à l’Actuel : Culture, Environnement, et Anthropologie (PACEA), Université Bordeaux, Talence, France and Centre for Early Sapiens Behaviour (SapienCE), Department of Archaeology, History, Cultural Studies and Religion, University of Bergen, Postboks 7805, 5020 Bergen (Norway)

[francesco.derrico@u-bordeaux.fr](mailto:francesco.derrico@u-bordeaux.fr)

**Table of Contents**

Supplementary Results A – Kolmogorov-Smirnov analysis

The materials, methods and results of the Kolmogorov-Smirnov analysis used to assess the differences in the distribution.

Supplementary Results B – Principal Coordinate Analysis

Results of the first and third axes of the regional Principal Coordinate Analysis of bead-type association recorded at Aurignacian and Gravettian sites.

Supplementary Results C – Archaeological Similarity Network analysis

Materials, methods, and results of the additional network, along with the summary and interval, and Similarity Radius index for the networks presented in the main text.

Supplementary Results D – Cluster Analysis

Materials, methods and results of the Cluster Analysis using detection algorithms to validate the visually identified clusters discussed in the main article networks.

#### **Supplementary Results A - Kolmogorov-Smirnov analysis**

We applied a Kolmogorov-Smirnov test to statistically assess variation in the number of ornament types across different site contexts. When applied to multiple groups, this test measures the maximum vertical difference (D) (i.e., the largest disparity between counts) between cumulative distribution functions, providing insights into the similarity or dissimilarity of distributions, with significance assessed by a p-value (Kolmogorov, 1933; Smirnov, 1948). Supplementary Figure 1 presents graphical representations of these distributions where the significant difference between Aurignacian and Gravettian occupations can be clearly visible. We also provide the values and graphical representation of these distributions when all the human remains found at the same site are treated as single analytical units (Supplementary Figure 2 & Supplementary Table 1).


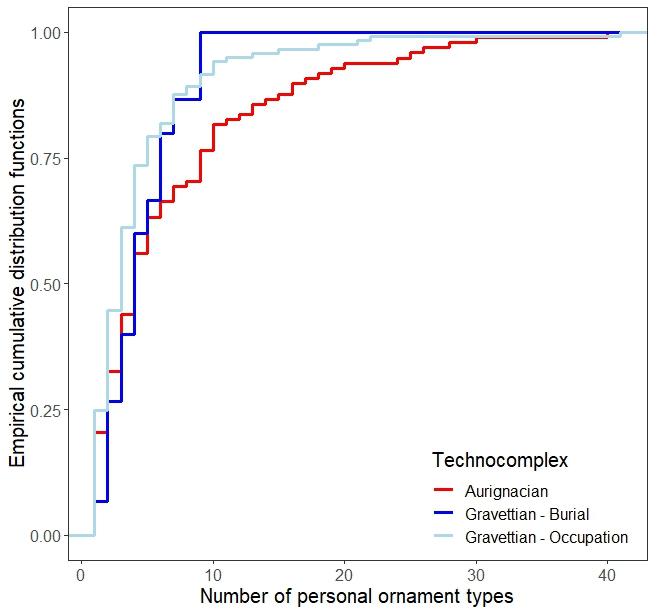


*Supplementary Figure 1 – Kolmogorov-Smirnov analysis of the distributions of the number of personal ornaments by site in each of the types of sites (Gravettian burials combined).*


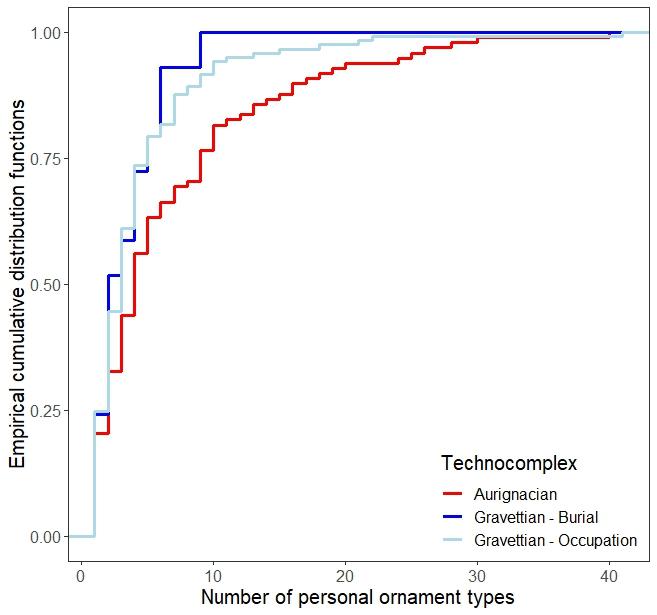


*Supplementary Figure 2 – Kolmogorov-Smirnov analysis of the distributions of the number of personal ornaments by site in each of the types of sites (Gravettian burials individual).*

|  | D Value | P-value |
| --- | --- | --- |
| Aurignacian and Gravettian occupations | 0.18848 | 0.0132 |
| Aurignacian occupations and Gravettian burials | 0.23469 | 0.2708 |
| Gravettian occupations and Gravettian burials | 0.21157 | 0.2872 |

*Supplementary Table 1– Results of the Kolmogorov-Smirnov test calculated for the bead-type associations recorded at Aurignacian occupation and Gravettian occupation and burial sites (Gravettian burials combined).*

#### **Supplementary Results B - Principal Coordinate Analysis**

In addition to analysing the first and second axes of the PCoAs for bead-type associations at Aurignacian and Gravettian sites across different European regions, we also examined the first and third axes to determine whether if the patterns initially observed persisted across other dimensions.

The first and third axes of the regional PCoAs, like those of the first and second axes, reveal a partial overlap in bead-type associations within each region (Supplementary Figure 3). The PCoA of sites from both North and South Iberia further demonstrates a substantial overlap in bead-type similarity (Supplementary Figure 4).


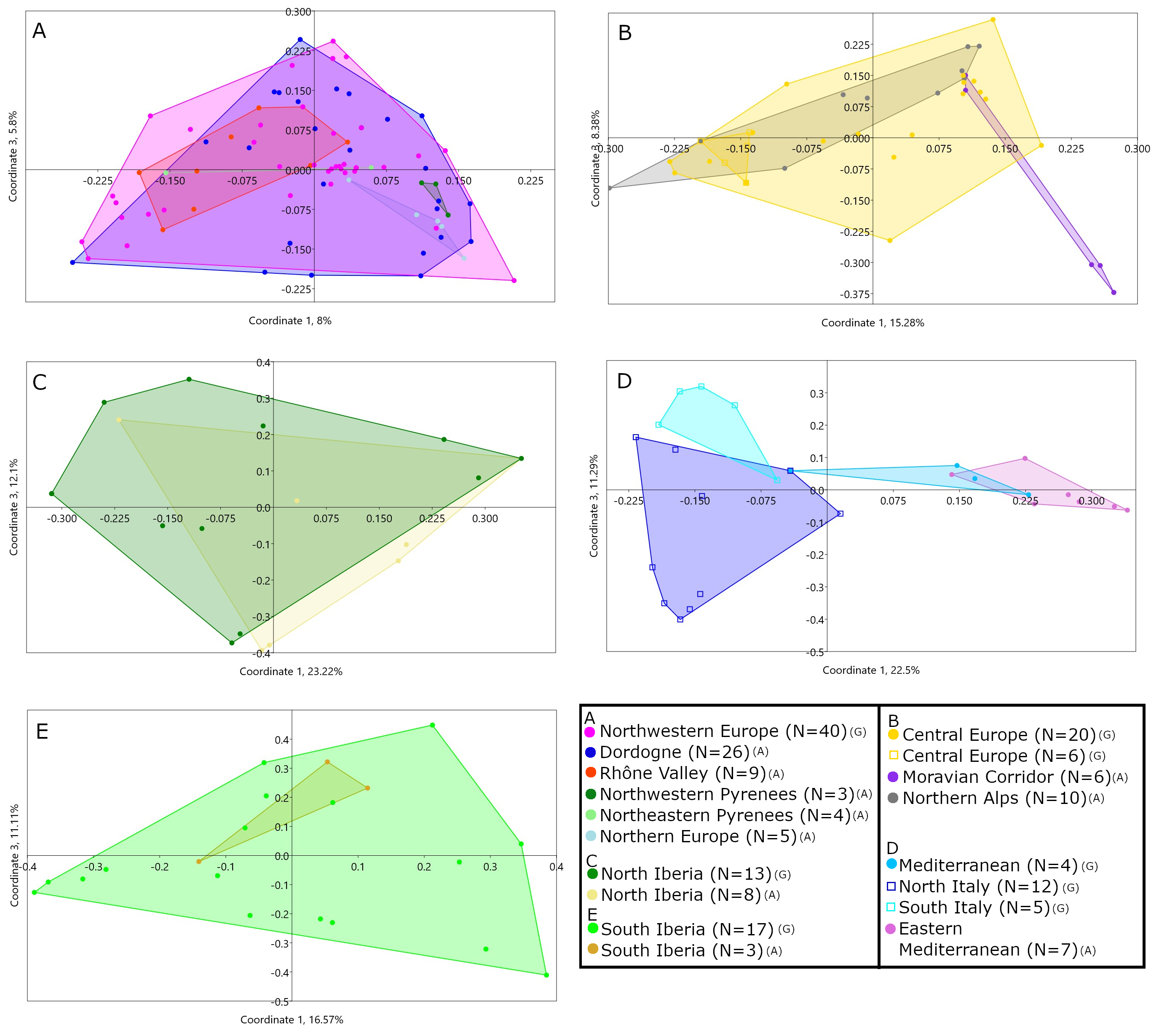


*Supplementary Figure 3 – First and third axes of the Principal Coordinate Analysis of bead-type association recorded at Aurignacian and Gravettian sites in a) North-western Europe, b) central Europe, c) north Iberia, d) western Mediterranean and e) south Iberia. Circle = occupation, square = burial. (A) = Aurignacian, (G) = Gravettian.*


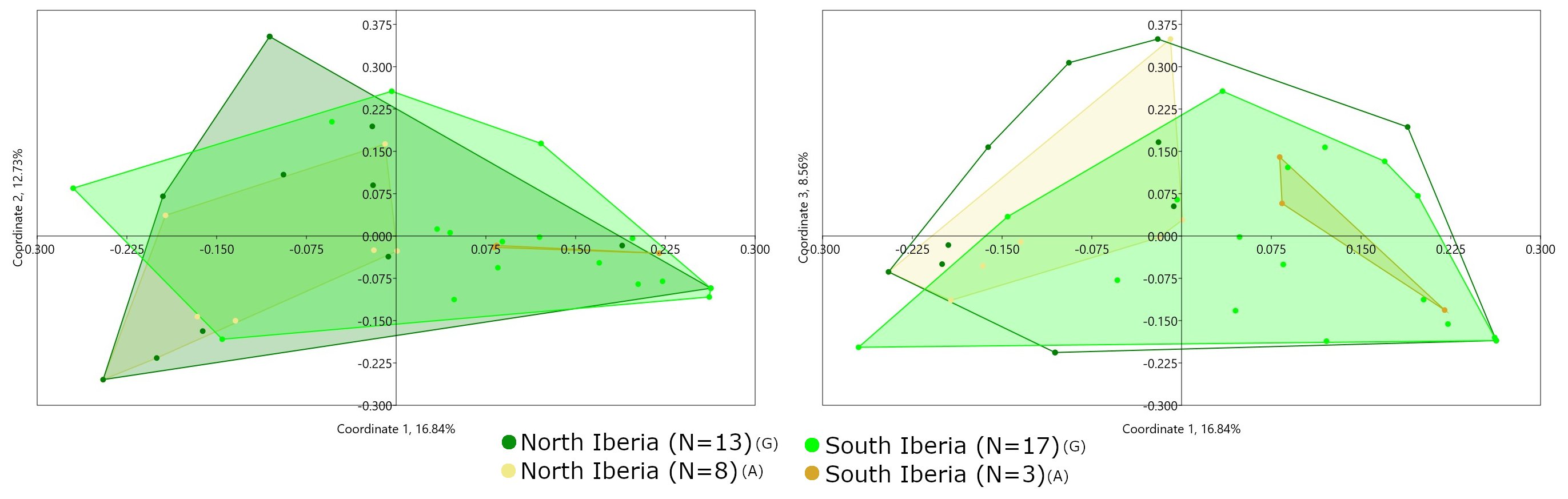


*Supplementary Figure 4 – First, second and third axes of the Principal Coordinate Analysis of bead-type association recorded at Aurignacian and Gravettian sites in north Iberia and south Iberia. (A) = Aurignacian, (G) = Gravettian.*

#### **Supplementary Results C - Archaeological Similarity Network analysis**

We applied Archaeological Social Network analysis to investigate the connections between sites attributed to the Aurignacian and the Gravettian based on shared personal ornament types. The two networks showing each technocomplexes separately (Figure 8) are colour-coded by regional group. However, these networks do not show connections between sites belonging to the other technocomplex. To address this topic, we also generated a network with both technocomplexes in a single graph (Figure 9), revealing connections between sites across both periods. However, this visualisation only indicates a site’s attribution to one of the two complexes, without showing its regional group. To provide a more comprehensive view, we created an additional version of this network that displays both the regional groupings and the connections between sites across technocomplexes (Supplementary Figure 5).


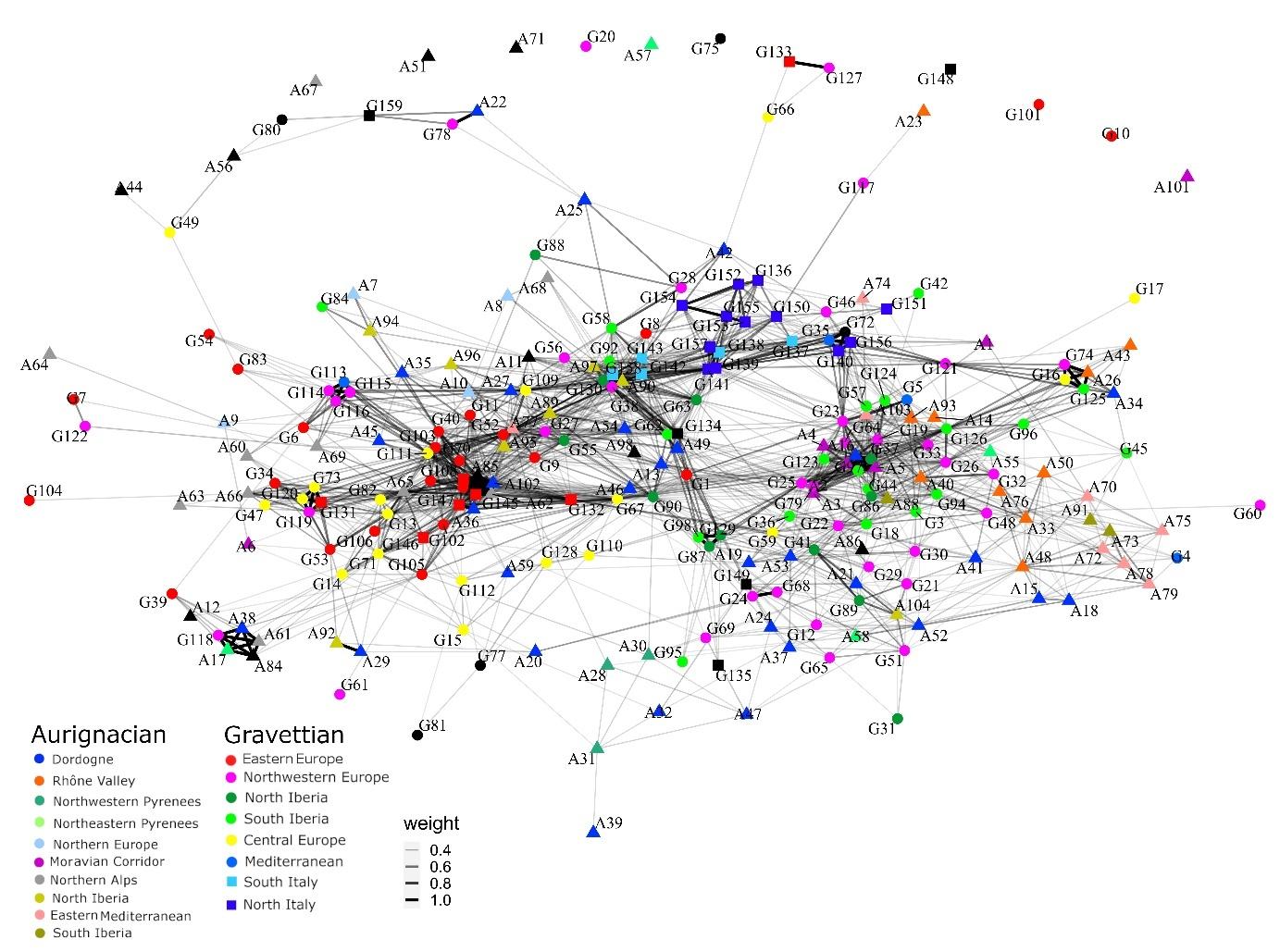


*Supplementary Figure 5 – Archaeological Similarity Network for Aurignacian and Gravettian full dataset showing regional groupings for each technocomplex plotted with Fruchterman-Rheingold layout. The ASNs’ edge weight (thickness) caption to the right of the corresponding network corresponds to the pairwise similarity value between connected nodes. Black symbols indicate sites not belonging to a set. Symbol code: triangle – Aurignacian; circle – Gravettian occupation; square – Gravettian burial.*

The cluster at the right of the graph (Supplementary Figure 5) is primarily composed of Aurignacian sites from the Rhône Valley (France), the eastern Mediterranean and Gravettian sites from north-western Europe and the Iberian Peninsula. The cluster at the left of the graph is composed principally of Aurignacian sites from the Dordogne (France) and from the south of the Iberian Peninsula, and Gravettian sites from central and eastern Europe. The final cluster, to the centre-top of the graph, is composed mostly of Aurignacian sites from the Dordogne and the north-western Pyrenees, and Gravettian burials from the north and south of Italy.

We also calculated the summary statistics for all the networks presented in the main text (Supplementary Table 2) using code adapted from Pereira et al. (2023). The results show that the network density is higher in the Gravettian network than in the Aurignacian network. All network interval statistics are provided in Supplementary Table 3. Additionally, the average similarity in personal ornament assemblages between sites is slightly higher in the Gravettian than in the Aurignacian (Supplementary Tables 3).

| **Network** | **Nodes** | **Edges** | **Threshold** | **Network Density** |
| --- | --- | --- | --- | --- |
| **Aurignacian** | 98 | 259 | 0.2 | 0.0544919 |
| **Gravettian** | 150 | 851 | 0.2 | 0.07615213 |
| **Aurignacian and Gravettian** | 248 | 1817 | 0.2 | 0.0593248 |

*Supplementary Table 2 – Individual Aurignacian and Gravettian ASN’s summary statistics.*

|  |  | **Max** | **Mean** | **Min** |
| --- | --- | --- | --- | --- |
| **Full** | Jaccard similarity index | 1.000 | 0.3562653 | 0.2058824 |
|  | Geographical index | 5486.285 | 1396.967 | 0 |
| **Aurignacian** | Jaccard similarity index | 1.000 | 0.3418912 | 0.2058824 |
|  | Geographical index | 4716.742 | 988.152 | 0 |
| **Gravettian** | Jaccard similarity index | 1.000 | 0.3651396 | 0.2093023 |
|  | Geographical index | 5486.285 | 1504.066 | 0 |

*Supplementary Table 3 – Aurignacian and Gravettian full and individual ASN’s network interval statistics. Geographical distance presented in kilometers, converted from Euclidean distance using the formula D * 111.319 (E Maria et al 2020)*

|  | Maximum | Mean | Minimum |
| --- | --- | --- | --- |
| Aurignacian | 19333.93 | 3212.134 | 0 |
| Gravettian | 22970.02 | 4773.782 | 0 |

*Supplementary Table 4 – Similarity Radius index for Aurignacian and Gravettian in full ASN (mathematical Infinite results removed).*

#### **Supplementary Results D - Cluster Analysis**

In the Archaeological Similarity Network (ASN) analysis presented in the main text, we visually identified several clusters within each network (Figures 8 and 9). To validate these clusters, we applied the Louvain Modularity algorithm (Dugué and Perez, 2015) to each network. Unlike visual identification, which does not impose strict boundaries or predefined sets of nodes within each cluster, the Louvain Modularity algorithm precisely determines both the number of clusters and their constituent nodes. Given these methodological differences, we validated our visually identified clusters by assessing whether the core group of nodes in each visually identified cluster was assigned to a single cluster by the algorithm. The algorithm was implemented using code adapted from Brughmans and Peeples (2023) as utilised in Pereira et al. (2023).

**Results**

The clusters identified by the Louvain Modularity algorithm are presented in the respective networks (Supplementary Figures 6, 7, and 8) as groups of nodes differentiated by colour. The algorithm identified at least seven clusters within the main compartment of each network. Upon analysing these algorithm-defined clusters, we found that in all three ASNs, the core groups of nodes in our visually identified clusters consistently corresponded to distinct clusters identifies by the algorithm. This alignment validates our visual cluster identification.


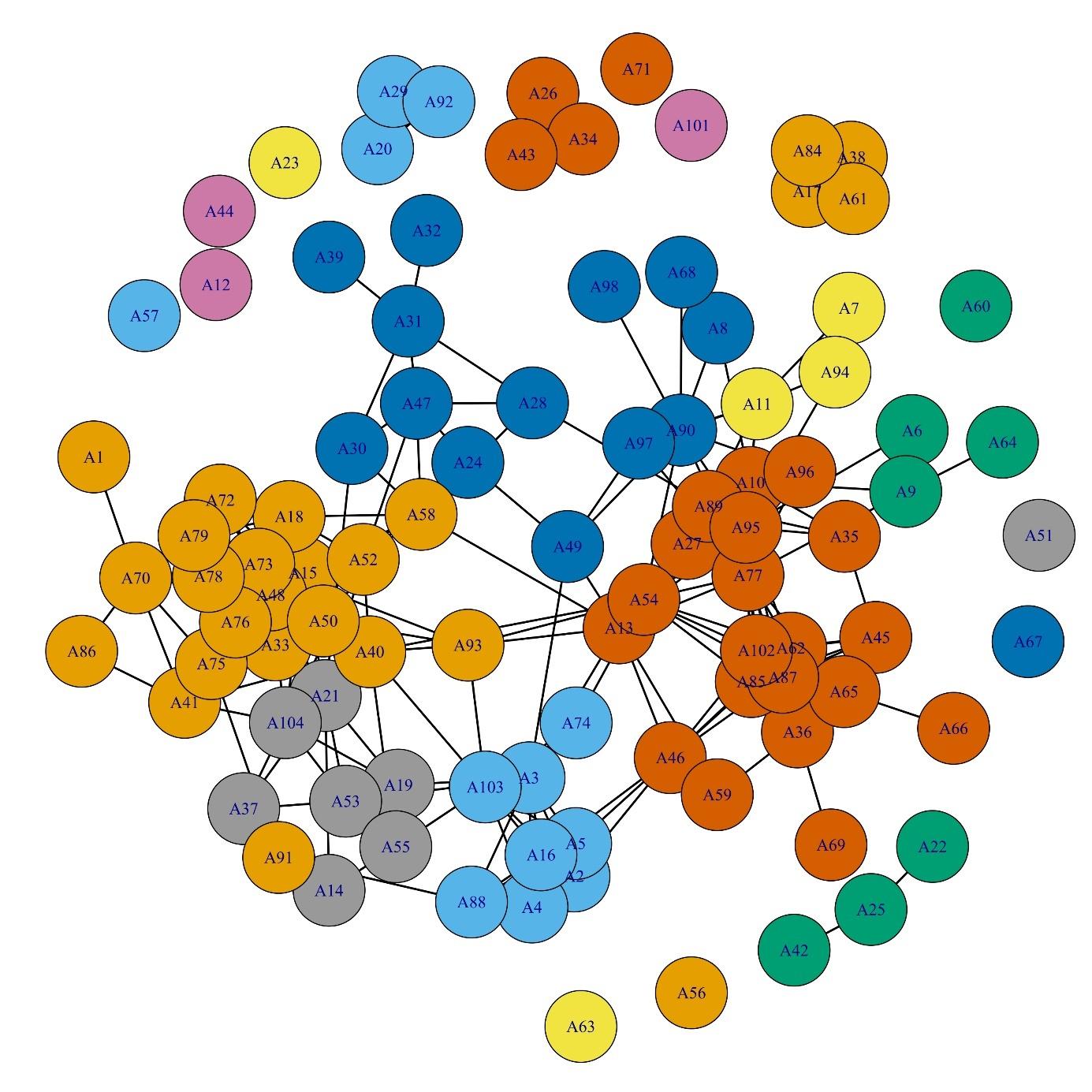


*Supplementary Figure 6 – Clustering analysis of the Aurignacian network (threshold = 0.2) using the Girvan-Newman cluster detection algorithm. Colours correspond to algorithm-identified*  *clusters.*

*
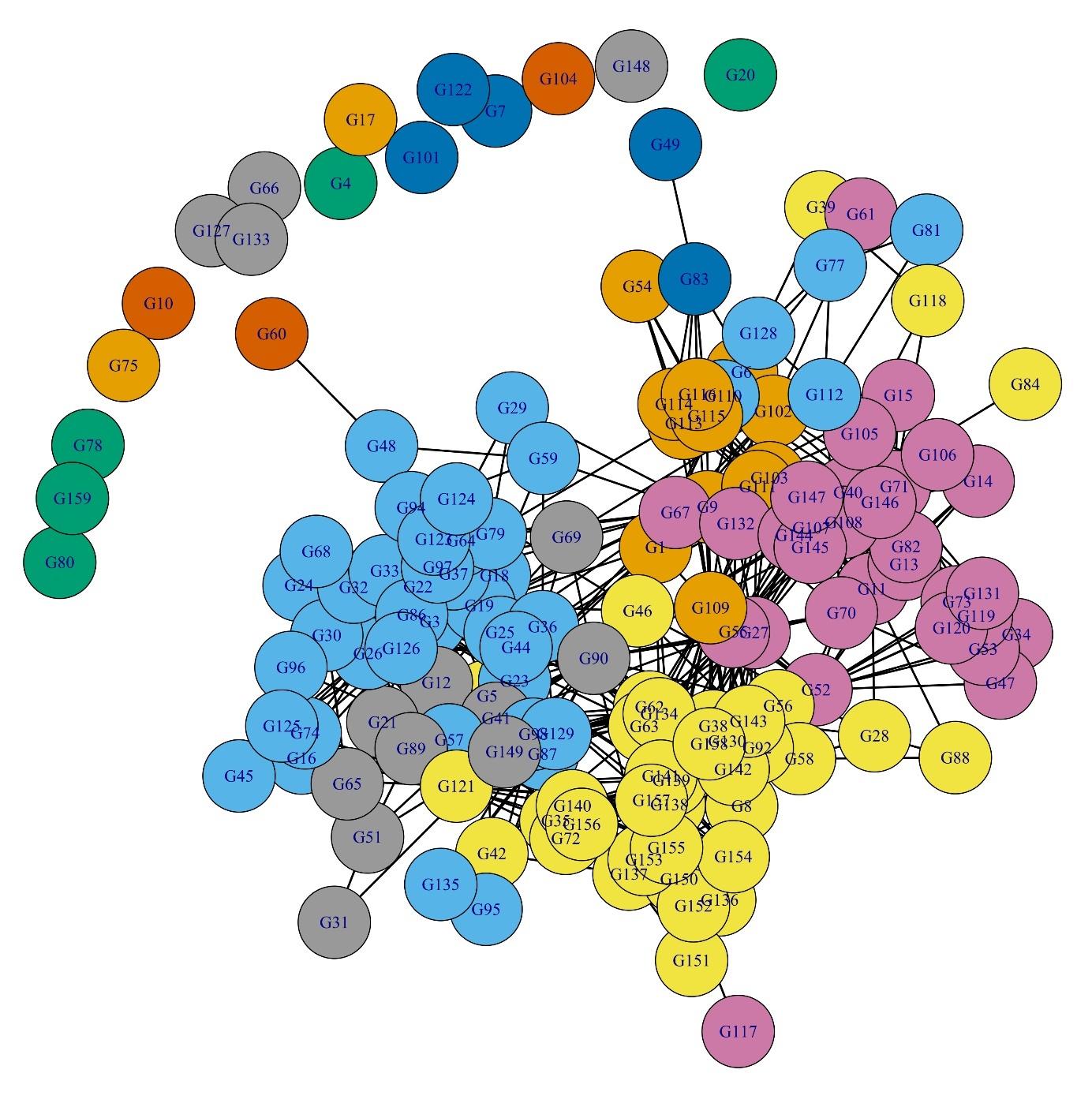
*

*Supplementary Figure 7 – Clustering analysis of the Gravettian network (threshold = 0.2) using the Girvan-Newman cluster detection algorithm. Colours correspond to algorithm-identified*  *clusters.*


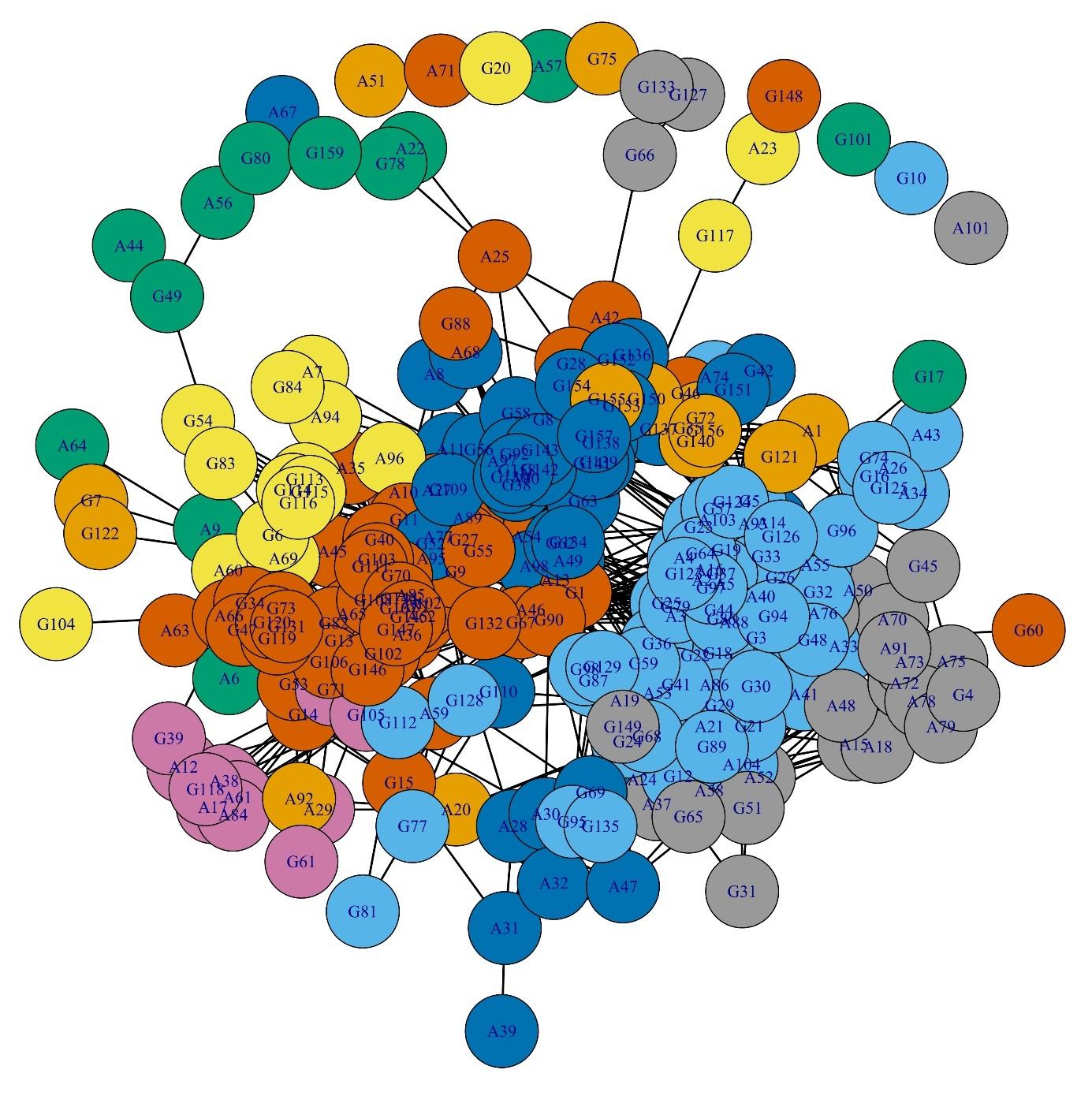


*Supplementary Figure 8 – Clustering analysis of the Combined network (threshold = 0.2) using the Girvan-Newman cluster detection algorithm. Colours correspond to algorithm-identified*  *clusters.*

#### **References**

Brughmans, T., Peeples, M.A., 2023. Network Science in Archaeology. Cambridge University Press.

Dugué, N., Perez, A., 2015. Directed Louvain: maximizing modularity in directed networks (PhD Thesis). Université d’Orléans.

Kolmogorov, A., 1933. Sulla determinazione empirica di una legge didistribuzione. Giorn Dell’inst Ital Degli Att 4, 89–91.

Maria E., Budiman E., Haviluddin, Taruk ., 2020. Journal of Physics. Conf. Ser. 1450 012080, doi:10.1088/1742-6596/1450/1/012080

Pereira, D., Manen, C., Rigaud, S., 2023. The shaping of social and symbolic capital during the transition to farming in the Western Mediterranean: Archaeological network analyses of pottery decorations and personal ornaments. Plos one 18, e0294111.

Smirnov, N., 1948. Table for estimating the goodness of fit of empirical distributions. The annals of mathematical statistics 19, 279–281.
